# Biosurfactants’ multifarious functional potential for sustainable agricultural practices

**DOI:** 10.3389/fbioe.2022.1047279

**Published:** 2022-12-12

**Authors:** Bhoomika M. Karamchandani, Ameya A. Pawar, Sujit S. Pawar, Sahil Syed, Nishigandha S. Mone, Sunil G. Dalvi, Pattanathu K. S. M. Rahman, Ibrahim M. Banat, Surekha K. Satpute

**Affiliations:** ^1^ Department of Microbiology, Savitribai Phule Pune University, Pune, Maharashtra, India; ^2^ Tissue Culture Section, Vasantdada Sugar Institute, Pune, India; ^3^ Discovery, School of Pharmacy and Biomolecular Sciences, Liverpool John Moores University, Liverpool, United Kingdom; ^4^ School of Biomedical Sciences, Faculty of Life and Health Sciences, University of Ulster, Coleraine, United Kingdom

**Keywords:** agriculture, biosurfactant/s, degradation, pollutants, pesticides, phytopathogens

## Abstract

Increasing food demand by the ever-growing population imposes an extra burden on the agricultural and food industries. Chemical-based pesticides, fungicides, fertilizers, and high-breeding crop varieties are typically employed to enhance crop productivity. Overexploitation of chemicals and their persistence in the environment, however, has detrimental effects on soil, water, and air which consequently disturb the food chain and the ecosystem. The lower aqueous solubility and higher hydrophobicity of agrochemicals, pesticides, metals, and hydrocarbons allow them to adhere to soil particles and, therefore, continue in the environment. Chemical pesticides, viz., organophosphate, organochlorine, and carbamate, are used regularly to protect agriculture produce. Hydrophobic pollutants strongly adhered to soil particles can be solubilized or desorbed through the usage of biosurfactant/s (BSs) or BS-producing and pesticide-degrading microorganisms. Among different types of BSs, rhamnolipids (RL), surfactin, mannosylerythritol lipids (MELs), and sophorolipids (SL) have been explored extensively due to their broad-spectrum antimicrobial activities against several phytopathogens. Different isoforms of lipopeptide, viz., iturin, fengycin, and surfactin, have also been reported against phytopathogens. The key role of BSs in designing and developing biopesticide formulations is to protect crops and our environment. Various functional properties such as wetting, spreading, penetration ability, and retention period are improved in surfactant-based formulations. This review emphasizes the use of diverse types of BSs and their source microorganisms to challenge phytopathogens. Extensive efforts seem to be focused on discovering the innovative antimicrobial potential of BSs to combat phytopathogens. We discussed the effectiveness of BSs in solubilizing pesticides to reduce their toxicity and contamination effects in the soil environment. Thus, we have shed some light on the use of BSs as an alternative to chemical pesticides and other agrochemicals as sparse literature discusses their interactions with pesticides. Life cycle assessment (LCA) and life cycle sustainability analysis (LCSA) quantifying their impact on human activities/interventions are also included. Nanoencapsulation of pesticide formulations is an innovative approach in minimizing pesticide doses and ultimately reducing their direct exposures to humans and animals. Some of the established big players and new entrants in the global BS market are providing promising solutions for agricultural practices. In conclusion, a better understanding of the role of BSs in pesticide solubilization and/or degradation by microorganisms represents a valuable approach to reducing their negative impact and maintaining sustainable agricultural practices.

## Introduction

Agriculture, food, and food processing industries are under great pressure to serve the huge food demands for an ever-growing population. Therefore, it is important to implement numerous effective strategies to improve crop provisions and yields. Some of the routinely practiced approaches include the use of harmful or harsh agrochemicals, fertilizers, and quick-breeding crop varieties. Several agrochemicals are usually utilized because of their bactericidal, fungicidal, insecticidal, herbicidal, and nematicidal activities. Important commercial crops such as wheat, rice, maize, and sorghum (staple foods), as well as sugarcane (cash crop), soybean, groundnuts, cotton, fruits, and vegetables are attacked by several pathogens and pests. Chemical origin organophosphate (profenofos, chlorpyrifos, and glyphosate), organochlorine (lindane, mirex, and chlordane), and carbamate-based pesticides are applied routinely in agriculture to control the growth of pests (Lamilla et al., 2021; Mdeni et al., 2022). Globally, ∼ two million tons of pesticides are used per year, 50% of which includes herbicides, 30% insecticides, 18% fungicides, and approximately 2% rodenticides and nematicides (Sharma et al., 2019; Sarkar et al., 2021). Organophosphorus and organochlorine-based pesticides are not specific toward the target and also remain in the ecosystem adhering to the soil as well as water bodies. The indiscriminate usage of chemical pesticides have harmful implications not only on soil health, fertility, microbial diversity, and the environment but also on human and animal health. Morbidities such as cancer, pulmonary dysfunction, and immune system deficiencies are frequently linked to these practices (Gupta, 2004; Sabarwal et al., 2018; Rani et al., 2021).

Huge quantities of pesticide residues are found in milk, meat, and other food products (Gill et al., 2020). Boedeker et al. (2020) reported ∼ 44% of the farming population is poisoned annually because of these synthetic chemicals. The cancerous, teratogenic, and immunosuppressive effects of agrochemicals and pesticides are of serious concern to human health (Choudhary et al., 2018). Most organophosphate pesticides are considered as class II, representing a moderately perilous pesticide where their acute toxicity (oral and dermal) to rats has been reported. They fall under this category due to their acute health risk as well as carcinogenic effects on humans and other animals (WHO, 2019^1^; Bhatt et al., 2021). Other factors such as climate change, environmental conditions, and pesticide resistance can also lead to the emergence of more aggressive pathogens and reduce the productivity of agricultural produce (Velásquez et al., 2018; Ma et al., 2021). Pesticides also adversely affect the natural beneficial flora and fauna (Khatoon et al., 2020) and obviously impact human wellbeing. The abuse of agrochemicals is subsequently impairing the agriculture sector and pesticide poisoning has become a serious concern for the past several decades (Aktar et al., 2009; Nicolopoulou-Stamati et al., 2016). The European Commission aims to reduce pesticide pollution up to 50% by 2030 (Business Today, 2022^2^). However, the global agricultural usage of pesticides has been increasing gradually from 1990 to date.

Several physical, chemical, and biological approaches are practiced in solubilizing and/or degrading harmful chemicals. Physical methods include soil washing, adsorption, electro-kinetic, granular-activated carbon, membrane filtration, and photocatalytic remediation. Chemical-based processes includes precipitation, floatation, ion exchange, coagulation, and flocculation methods. Most of the routinely used physicochemical methods are comparatively less cost-effective and not eco-friendly. Occasionally, combinations of physicochemical methods work successfully (Akhtar et al., 2020). Currently, many pesticide formulations available on the market is comprised of synthetic surfactant as adjuvants (Castro et al., 2013). Surfactants with oil-based adjuvants in chemical-based formulations mainly provide improved adhesion of pesticide to plant surfaces for pesticides to plant surfaces and parts, in addition to other functional properties such as enhanced wetting, spreading, penetration, and retention periods (Roehrig et al., 2018). Other well-known properties of surfactants include reduction in surface tension (ST) and interfacial tension (IFT) that broaden their applications as emulsifying, dispersing, solubilizing, foaming, and wetting agents for the agriculture sector (Sarubbo et al., 2022). The formulations for application purposes can be modified by the amalgamation of emulsions with water-soluble adjuvants. Spray adjuvants are generally useful for application of pesticide in order to enhance their effectiveness against pests and diseases (Prado et al., 2016). Surfactants are indispensable for agrochemical-based formulations, to improve their performance, and uphold their stability. Surfactants facilitate the foliar uptake of herbicides, defoliants, and growth regulators (Castro et al., 2013).

In addition to the aforementioned attributes, biosurfactants (BSs) of microbial/plant origin possess functional properties such as substantial antimicrobial activity, biodegradability, and eco-friendly nature, and are therefore, quite advantageous for agrochemical formulations (Marchant and Banat, 2012a). Along with their surface-active properties, BSs also exhibit noticeable pesticidal and antimicrobial properties (Fracchia et al., 2015; Ceresa et al., 2021). This proves them as a prime candidate for leading the path toward the sustainable management of agricultural pests and pathogens and avoiding the use of chemical pesticides (Naughton et al., 2019; Kumar et al., 2021; Karamchandani et al., 2022a; Karamchandani et al., 2022b; Adetunji et al., 2022; Sánchez, C., 2022). BSs are promising suitable and sustainable alternatives to chemical or synthetic surfactants mainly due to their lower toxicity to the human system (Marchant and Banat, 2012b). Overall environmental health can be improved by employing BSs in various industrial production processes (Manga et al., 2021). The presence of hydrophilic and hydrophobic moieties facilitates interactions between immiscible liquids of agricultural formulations which can be challenging in nature. Therefore, understanding the role of BSs as “biopesticides” presents huge opportunities in the global surfactant market (Karamchandani et al., 2022a; Karamchandani et al., 2022b). Biological and eco-friendly approaches involving microorganisms, especially plant growth-promoting rhizobacteria (PGPR) and other autochthonous microbes, are often helpful in breaking down pollutants and converting them into non-toxic or low-toxic metabolites (Gayathiri et al., 2022). Sustainable agricultural strategies also include the use of biomaterials such as chitin and chitosan (CH) in agricultural formulations to replace the need for harsh chemicals (Karamchandani et al., 2022a; Karamchandani et al., 2022b).

This review article aims to collate and present up-to-date information on the role of BSs in combating phytopathogens associated with several commercially valued crops. The current article also discusses the efficiency of BSs in the solubilization of pesticides to reduce their toxicity and contamination of soils. Assessment methodologies such as the life cycle assessment (LCA) and life cycle sustainability analysis (LCSA) are also presented to highlight their social, environmental, and economic impacts. An innovative approach of nanoencapsulation for pesticide formulations is discussed for minimizing pesticide doses and reducing their direct exposure to humans and animals. In addition, we examine the role of nanotechnology and the amalgamation of BSs with other nano-biomaterials for sustainable agricultural applications. Finally, we discuss the novelty of some established industries and newer applicants involved in creating a prominent impact on BS production and sustainable agricultural practices.

## Use of biosurfactants and/or BS-producing microorganisms in combating phytopathogens

**FIGURE 1:**
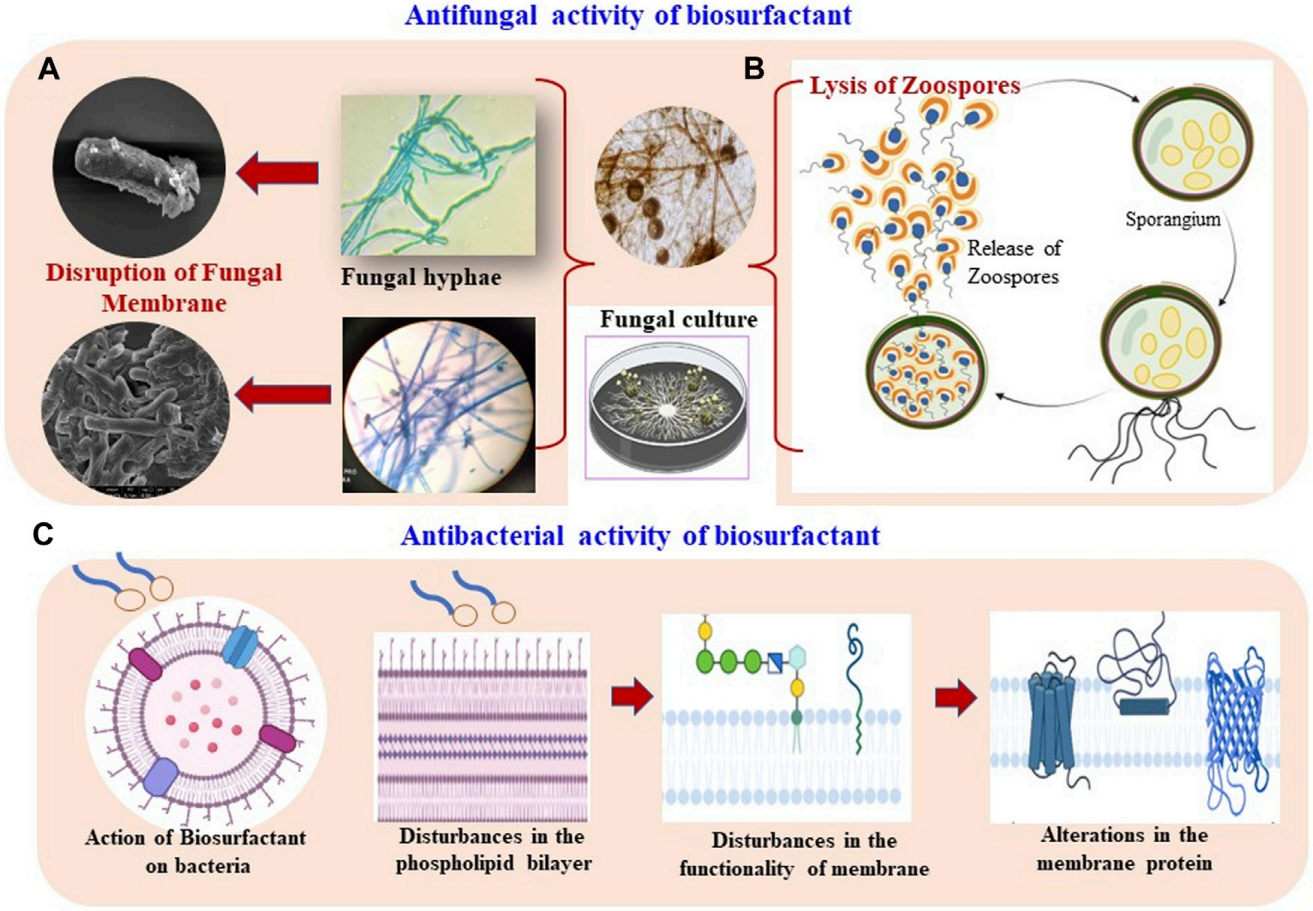
Antimicrobial potential of biosurfactants. **(A)** Distortion of fungal mycelia, **(B)** lysis of fungal zoospore, and **(C)** antibacterial action of biosurfactants by disturbing membrane permeability.

Over the past few decades, plant pathogens have been causing serious damage to crops before and/or after harvest, resulting in 10–40% crop yield losses and eventually leading to economic deficit (Savary et al., 2019). A wide range of foods including cereals (rice, wheat, maize, sorghum, millets–rye, barley, soybeans, oats, and teff), fruits, vegetables, or starchy tubers (potatoes, sweet potatoes, yams, and cassava) are indispensable components of our day-to-day lives. Various chemicals are used to protect perishable fruits, seeds, flowers, and foliage at different phases of the plant’s growth. The extensive use of chemicals undoubtedly has a negative impact not only on soil health but also on plant growth. Such challenges can be addressed through BS-based eco-friendly solutions to eradicate or reduce the load of plant pathogens (Adnan et al., 2018; Chopra et al., 2020a; Karamchandani et al., 2022a). A variety of BSs, namely, rhamnolipid (RL), surfactin, mannosylerythritol lipids (MELs), sophorolipids (SL), trehalose lipids (TL), and cellobiose lipids (CL) have been described to have antimicrobial activity against phytopathogens (bacteria, fungi, viruses, insects, and larvae) (Díaz De Rienzo et al., 2016; Valotteau et al., 2017; Kumar et al., 2021; Handore et al., 2022). Among BSs, RL, surfactin, and MELs are well-documented to have a broad-spectrum antimicrobial activity. Diverse types of BSs are well-known for their broad-spectrum antimicrobial activity against pathogens associated with foods and agricultural produce. The overall antimicrobial activity of BSs against phytopathogens is shown in Figure 1.

Many BS-producing microorganisms and PGPR have been utilized in agriculture as bioinoculants or biofertilizers due to their metabolically versatile nature. PGPR represent indigenous microbial communities that are valuable in promoting plant growth and defeating phytopathogens. Substantial literature discusses the positive effect of PGPR on plant growth through the enhanced production of phytohormones, enabling nutrient uptake, nitrogen fixation, and solubilization of inorganic phosphates in the soil (Chopra et al., 2020a; Chopra et al., 2020b). Several antagonistic compounds synthesized by PGPR restrict the proliferation of phytopathogens. PGPR strains typically confer traits such as solubilization of phosphate, production of indole acetic acid (IAA), siderophores, catalase, and NH_3_ (Lal et al., 2018). Microorganisms in the rhizospheric ecosystem produce various metabolites including BSs, showing significant biocontrol potential (Arifiyanto et al., 2020). Table 1 shows the applications of different types of BSs in controlling varied phytopathogens.

**TABLE 1:** Agriculture-related applications of biosurfactants in controlling phytopathogens and bioremediation.

Type of BS	BS-producing organism and its source	Antimicrobial activity of BS against phytopathogens	Crops affected by the phytopathogen/s	Applications of BS/s or producing organism	References
Rhamnolipid (mixture of mono- and di-rhamnolipid)	Commercial source (AGAE Technologies, United States)	*Xanthomonas campestris* NCIM 5028, *Fusarium moniliforme* ITCC 191, *F. moniliforme* ITCC 4432, *F. graminearum* ITCC 5334	• Citrus fruits• Wheat• Sugarcane	• Synergistic activity demonstrated for rhamnolipid biosurfactants and nanoparticles derived from fungal origin chitosan against bacterial and fungal phytopathogens	Karamchandani et al. (2022a)
Mixture of various mono- and di-rhamnolipids	*Pseudomonas guariconensis* LE3 (*Lycopersicon esculentum* rhizosphere soil)	*Macrophomina phaseolina* causes charcoal rot disease in sunflower	Sunflower (*Helianthus annus*)	• Strain LE3 has plant growth-promoting traits• Biocontrol activity of BS and LE3 against charcoal rot disease• Isolate LE3 is an ideal candidate for BS production and BS-based bioformulation to maximize agricultural produce and sustainability	Khare and Arora (2021)
Rhamnolipid (predominance of di-RL congeners (85%) with an abundance of hydroxydecanoyl-hydroxyde-canoate	*Pseudomonas aeruginosa*	N/A	N/A	• High extraction capacity for arsenic, cadmium, and zinc, demonstrating applications for bioremediation purpose• Harmless to commensal soil bacteria• Biocompatibility with *Artemia salina* (eukaryotic bioindicator)• Recycling of metal contaminants and strengthening applications of BSs in soil remediation• Reducing the costs of mining activity	Lopes et al., (2021)
Mono- and di-rhamnolipid (di-rhamnolipid as major component)	*Pseudomonas aeruginosa* RTE4 (tea rhizosphere soil)	*Corticium invisium, F. solani X. campestris*	Tea	• Plant growth-promoting traits: production of indole acetic acid (IAA), hydrolytic enzymes, and solubilization of tri-calcium phosphate (bio-stimulant)• Biocontrol activity against tea plant pathogens	Chopra et al. (2020b)
N/A	*Bacillus cereus* (MG547975) (tomato rhizosphere soil)	*Fusarium oxysporum Alternaria solani*	Tomato	• Plant growth-promoting traits: production of IAA, ammonia, catalase, siderophore, and 1-aminocyclopropane-1-carboxylate deaminase, along with nitrogen fixation abilities• Bio-stimulant property• Biocontrol against tomato pathogens• Suppression of fungal growth through the production of lytic enzymes	Karthika et al. (2020)
N/A	*Bacillus subtilis* V26	*Botrytis cinerea, Tuta absoluta*	• Grape• Tomato	• Biocontrol:protection against grey mold• Bioinsecticide activity against larvae of *T. absoluta*	Khedher et al. (2020)
N/A	*Xylaria regalis* (*Thuja plicata* cones)	*F. oxysporum, Aspergillus niger*	Chilli	• Plant growth-promoting traits: production of IAA, hydroxamate-type siderophore, hydrogen cyanide, phosphate solubilization ability• Increase in chlorophyll, nitrogen, and phosphorous content after application of *X. regalis*	Adnan et al. (2018)
Rhamnolipid (mono- and di-rhamnolipid)	*Pseudomonas rhizophila* S211 (rhizosphere soil from a pesticide-contaminated artichoke field)	N/A	N/A	• Plant growth-promoting traits and remediating activity through the synthesis of ACC deaminase, putative dioxygenases, auxin, and pyroverdin• Production of BS and exopolysaccharide levan• Enhancement of pesticide solubilization	Hassen et al. (2018)
Fengycin-type lipopeptides	*Bacillus sp.* MA04	*Penicillium expansum, A. niger Colletotrichum sp. Diplocarpon rosae F. stilboides, Sclerotium rolfsii, Rhizoctonia solani*	• Tomato • Pepper• Mango• Apple• Rose	• Plant growth-promoting traits: production of IAA and ACC deaminase, phosphate solubilization• Antifungal activity• Emulsification ability—useful in formulating organic and chemical molecules as fertilizers and pesticides	Herna´ndez-Morales et al. (2018)
Lipopeptide (surfactin)	*Bacillus sp.* FJAT-14262 (*Anoectochilus roxburghii* rhizosphere soil)	*F. oxysporum*	N/A	• Biocontrol activity of BSs against fusarium wilt	Chen et al., (2017)
Sophorolipids	*Rhodotorula babjevae* YS3 (agricultural soil)	*Colletotrichum gloeosporioides* ITCC 6434, *F. verticilliodes* MTCC 10556, *F. oxysporum f. sp. Pisi* ITCC 4814, *Corynespora cassiicola* ITCC 6748, *Trichophyton rubrum* MTCC 8477	N/A	• Antifungal activity• BS stable over a range of pH, temperature, and salinity, demonstrating its application potential in environmental and industrial sectors	Sen et al., (2017)
Lipopeptide (a mixture of isoforms of surfactin, iturin, and fengycin)	*Bacillus subtilis* SPB1 (soil sample contaminated by hydrocarbons)	*R. bataticola, R. solani*	N/A	• Biocontrol activity against phytopathogenic fungi	Mnif et al., (2016)
Mannosylerythritol lipids	*Pseudozyma* sp	*Blumeria graminis* f. sp. *tritici* strain T-10	Wheat	• Biocontrol activity: suppressing powdery mildew of wheat• Reduces hydrophobicity of solid surfaces, proving their potential for applications in agricultural formulations	Yoshida et al. (2015)

N/A, not available.

**FIGURE 2:**
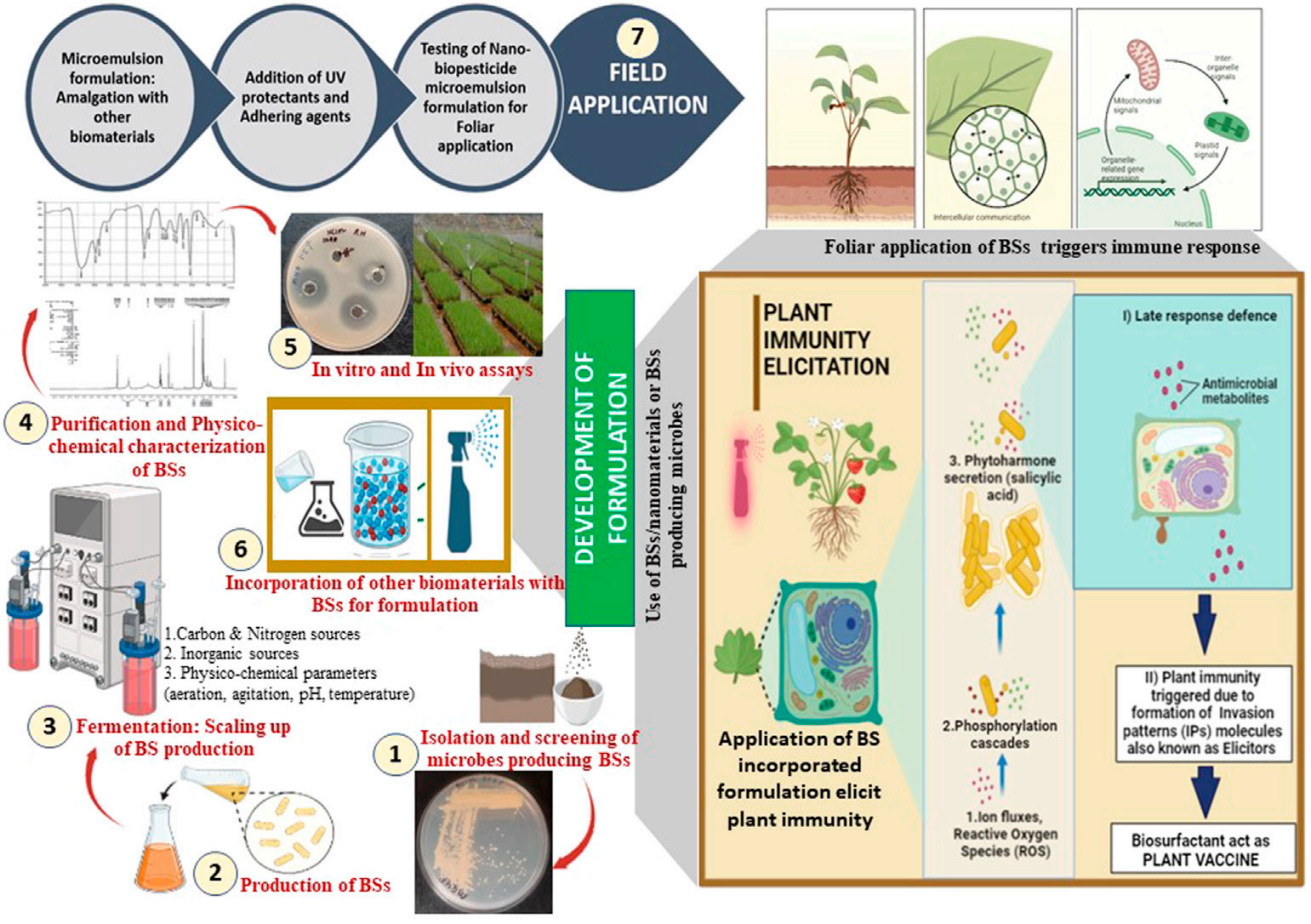
Application of a formulation incorporating biosurfactants (BSs) in the elicitation of plant immune response.

The agricultural sector has been actively looking for biocontrol strategies to satisfy food demands in a sustainable way. Versatile PGPR and BS-producing organisms, such as *Pseudomonas*, *Bacillus,* and *Burkholderia*, have been frequently reported from the rhizosphere of plants such as tea, tomato, and wheat (Villa-Rodriguez et al., 2019; Chopra et al., 2020b; Cochard et al., 2022). Root exudates of the rhizosphere provide a suitable niche harboring metabolically diverse PGPR communities due to the presence of nutritionally rich constituents such as carbohydrates, organic acids, minerals, vitamins, phenolics, and hydrocarbons. Organic farming can be well-established through the exploitation of microbial communities and their metabolites in the form of biocontrol agents for augmenting not only food safety but also crop protection. Recently, Chopra et al. (2020b) reported the PGPR strain *P. aeruginosa* RTE4 from the tea rhizosphere and its RL-BS for biocontrol activity against *Corticium invisium*, *Fusarium solani* (a foliar tea pathogen), and *Xanthomonas campestris* (citrus fruit pathogen). RL-BS derived from strain RTE4 has attractive physicochemical properties and can act as “bio-fungicide” similar to carbendazim—a commercial fungicide. Khare and Arora (2021) reported a novel PGPR and BS-producing *P. guariconensis* LE3 from the *Lycopersicon esculentum* (tomato) rhizosphere and demonstrated its potential for bioformulation. RL-BS (mixture—mono and di) exhibited antimicrobial activity against *Macrophomina phaseolina* which is one of the most widespread fungal pathogens causing charcoal rot, collar rot, damping-off, stem rot, and seedling blight in crops such as sunflower, soybean, sorghum, and groundnut. The strain LE3 posseses traits such as the solubilization of phosphate and production of siderophores, IAA, NH_3_, and 1-aminocyclopropane-1-carboxylate deaminase (ACCD). Molecular studies of *P. guariconensis* LE3 showed genes responsible for the synthesis of several metabolites (antibiotics, diacetylphloroglucinol, phenazine 1-carboxylic acid, and pyocyanin) and lytic enzymes (chitinase and endoglucanase) that are required in anti-phytopathogenic and biocontrol activities. Considering the beneficial properties of bacterium LE3, Khare and Arora (2021) designed a bioformulation amended with BSs that showed an enhanced yield (80.80%) of sunflower (*Helianthus annuus*) under laboratory and field conditions. The authors inoculated LE3 culture with BSs to the adhered soil mass of the root of plantlets and observed significantly improved biocontrol activity (75%) against *M. phaseolina*. A formulation designed with the cells of LE3 and BSs enhanced the yield and biocontrol activity of 75.45% in sunflower. The presence of BSs in the formulation promisingly facilitates the plant–bacterial interaction and also improves soil properties to control plant diseases. Thus, BS-based formulations are extremely beneficial in enhancing the overall health status of the plant, seedling growth, seed germination, and crop yield. Thus, Khare and Arora (2021) recommended designing an efficacious crop-specific formulation bearing remarkable features such as 1) high competency in the rhizosphere environment, 2) widespread saprophytic ability, 3) growth-enhancing or improving abilities, 4) effortlessness mass production processes, 5) broad-spectrum antimicrobial potential, 6) environmentally safe, and 7) biocompatibility with other associating living forms. Along with antimicrobial functionality, RL-BS plays an important role in stimulating plant immunity which subsequently reduces infections caused by phytopathogens (see Figure 2). RLs and lipopeptides attack pathogens through antimicrobial activity or stimulate the immune system in plants to protect them from disease conditions (Delaunois et al., 2014; Bardin et al., 2015; Keswani et al., 2019).

RL-type BSs exhibit significant antifungal activities through 1) lysis of zoospores, 2) their effect on spore germination, and 3) inhibiting mycelial growth. The amphiphilic nature of BSs allows them to access the fungal plasma membranes (Otzen, 2017), particularly zoospores, which lack a cell wall (Stanghellini and Miller, 1997). Thus, RLs can destabilize the mycelia of fungi and lyse them. The lipid composition of RLs influences the membrane partition process (Sánchez et al., 2007, 2010) through intercalation with phosphatidylcholine and phosphatidylethanolamine bilayers which alters their packing (Abbasi et al., 2012, 2013). The cell membranes possess phospholipids as a vital component that contributes toward signal transduction, storage of energy, and adaptability to several environmental conditions (Wang et al., 2019). RLs also disturb the functionality of the fungal cell membrane through structural distortions or perturbations in the physicochemical properties of the phospholipid bilayer, subsequently affecting the hydration status and diffusion dynamics of the water/lipid interface. The concentration of BSs in this regard is noteworthy and a certain concentration is required to form aggregates in the solution. Thus, RLs hold great potential for agriculture (Randhawa and Rahman 2014).

The biocontrol effect of RL-BS has also been tested on fruits and vegetables. Tomato and edible berries from *Solanum lycopersicum* L. are fragile and imperative vegetable crops which need to be protected from the vigorous activities of phytopathogens. Early blight is one of the most destructive disease that results in spotting with yellow coloration of tomato leaves. The small dark spots found in the initial stage develop into larger spots with a concentric ring structure. In the case of a severely affected tomato plant, leaves turn brown and fall off. *A*. *solani* is one of the dreaded fungi that causes early blight disease in tomato plants. Recently, Lahkar et al. (2015) investigated the effect of *P. aeruginosa* JS29-derived BSs on *A. solani* and reported inhibition in fungal growth by 73% at a concentration of 3.00 g/L under laboratory conditions. In a field trial study, BSs efficiently inhibited *A. solani* when they are used at a concentration of 1.50 g/L. For the first time, the authors demonstrated that RL-BS mediated complete inhibition of early blight disease in tomatoes caused by *A. solani* in a field-based study.

Several *Bacillus* strains, namely, *B. subtilis, B. pumilus, B. amyloliquefaciens, B. licheniformis,* and *B. mojavensis* are often reported for the production of surfactin—a cyclic lipopeptide BS. Lipopeptide isoforms such as iturin, surfactin, and fengycin have also been reported frequently. Hernandez-Morales et al. (2018) reported a BS (a mixture of fengycins A and B) from the *Bacillus* sp. MA04 PGPR strain of tomato rhizosphere. The fengycins had a strong antifungal activity (up to 97% inhibition) against *Penicillium expansum, F. stilboides, Sclerotium rolfsii, and R. solani* (Hernandez-Morales et al., 2018). The BS-producing *Bacillus* sp. MA04, being a PGPR, further supports plant health.

Mnif et al. (2016) extracted mixtures of surfactin isoforms (MW of 1,007 Da, 1,021 Da, and 1,035 Da), iturin isoforms (MW of 1,028 Da, 1,042 Da, and 1,056 Da), and fengycin isoforms (MW of 1,432 Da and 1,446 Da) along with two new un-identified lipopeptide clusters (MW of 1,410 Da, 1,424 Da, 973 Da, and 987 Da) from *B. subtilis* SPB1. The authors reported fungicidal activity of *B. subtilis* SPB1 origin lipopeptide isoforms against *R. bataticola* (MIC 0.04 mg/ml, IC 50% at 0.012 mg/ml, and IC 90% at 0.02 mg/ml) and *R. solani* (MIC:4 mg/ml, IC 50% at 0.25 mg/ml, and IC 90% at 3.3 mg/ml). The lipopeptide BS resulted in a loss of sclerotic integrity, hyphal shortening, and cell lysis of fungi suggesting promising biocontrol potential against pathogens responsible for disease conditions.

Tomato (*Solanum lycopersicum*) is an imperative vegetable both economically and nutritionally and is the 3rd largest crop after potato and onion worldwide. Tomato fruits possess unique properties and are a rich source of vitamins A and C and antioxidants. In addition to *A. solani*, other fungal pathogens, namely, *B. cinerea, A. alternata,* and *F. oxysporum* also affect the quality and quantity of tomato fruits significantly both in the pre- and post-harvesting stage. Among varied pathogens, *Fusarium*, *Pythium*, and *Rhizoctonia* cause root rot or damping-off and wilt which negatively affects the quality and yield of the tomato fruits (Kavroulakis et al., 2010; Lamichhane et al., 2017). A study by Karthika et al. (2020) showed the growth inhibition of *F. oxysporum* (causes vascular wilt in tomato) by 66% and *A. solani* (causes blight in tomato and potato) by 54% through the activities of a metabolically versatile BS-producing strain *B. cereus* KTMA4. This bacterium produced varied plant growth-promoting factors and was able to fix nitrogen. The BS-producing *Bacillus* strain can also produce biofilms and can tolerate salinity (5% NaCl). *In vivo* studies indicated an enhanced percentage of seed germination and vigor index when seeds of tomato were grown after treatment with KTMA4 BSs. Because of significant yield losses caused by fungal pathogens, new, efficient, and environmentally safe methods of pest control are needed, hence, amphiphilic compounds or the BSs produced by many microbes are considered a good alternative.

Galitskaya et al. (2022) reported the use of *B. mojavensis* P1709-derived lipopeptide BSs against *F. oxysporum* f. sp. *lycopersici* affecting the cherry tomato. The authors assessed the ability of BSs to protect post-harvested cherry tomatoes from decay and mycotoxin contamination caused by the fungal pathogen. The genome of *B. mojavensis* P1709 contained two genes (fend and srfAA), responsible for the synthesis of compounds (fengycin and surfactin families). The acid-precipitated fraction (APF) of the *B. mojavensis* P1709 culture medium inhibited radial growth (at a concentration of 20 g/L) of the fungal pathogen on agar plates by 93% and mycotoxin (T-2, HT-2) production by 98% after 5 days of growth. An *in vivo* test indicated that the APF successfully suppressed the fungal growth in and on cherry tomato fruits by 93% and 25% on the 2nd and 7th days of incubation, respectively. The results demonstrated the antifungal action of BSs produced by *B. mojavensis* P1709 for protecting post-harvest cherry tomatoes from fungal mold decay and mycotoxin contamination.

It is important to note that both plant growth-promoting and BS producers have not only been isolated from the rhizosphere of fruits and food crops but also from culinary and medicinal plants. *Anoectochilus roxburghii*—a commercial valued ornamental crop found in many Asian countries—has been used to isolate *Bacillus* sp. possessing PGPR traits (Chen et al., 2017). The authors extracted surfactin from *A. roxburghii* origin *Bacillus* sp. FJAT-14262 and reported its biocontrol activity against *F. oxysporum* (a causative agent of *Fusarium* wilt). The authors also reported structurally conserved non-ribosomal peptide synthetase genes srfAA, srfAB, and srfAC in the *Bacillus* sp. FJAT-14262 genome, responsible for surfactin peptide biosynthesis. Mejri et al. (2018) reported a reduction in disease severity caused by *Z. tritici* through foliar spray possessing myco-subtilin, surfactin, and fengycin at a concentration of 100 mg/L.

Khedher et al. (2020) described two biological activities of BSs produced by *B. subtilis* V26 against pathogens affecting grapes and tomatos. First, the antifungal activity of BSs against *Botrytis cinerea* resulted in swelling and deformation of the fungal hyphae, and second, insecticidal activity resulted in histological alterations or damage in the midgut of *Tuta absoluta* larvae (at LC50 = 278.78 ng/cm^2^). Thus, the antifungal and insecticidal activities of the BS allow its application potential as a biocontrol agent. Similar to vegetables, various fruits and cereal crops of commercial value are attacked by several phytopathogens. The fungus, *Colletotrichum gloeosporioides*, affects mango, papaya, citrus, and avocado, whereas *Phytophthora infestans* was reported to attack potato. An important plant pathogenic fungus *Sclerotinia sclerotiorum* shows a broad host range including oilseed rape and soybean. Goswami and Deka (2019) demonstrated the antifungal activity of *B. altitudinis* MS16-derived lipopeptide BSs. A mixture of surfactin and iturin inhibited the growth of *C. gloeosporioides* and *S. sclerotiorum* by approximately 42.8 and 41.2%, respectively. The strain *B. altitudinis* MS16 produced a BS (yield of 3.8 g/L) that reduced the ST from 72.8 to 32.3 mN/m and that has good emulsification abilities and stability.

SL-type BSs produced by yeast strains have also been evaluated for antifungal activity. Sen et al. (2017) isolated a novel yeast strain—*Rhodotorula babjevae* YS3—from an agricultural arena and demonstrated the production of heterogeneous type SL (yield of 19.0 g/L). The SL-BS reduced the SFT from 70 to 32.6 mN/m with a CMC of 130 mg/L and showed good oil spreading (38.46 mm^2^) capacity with 100% emulsifying activity against crude oil. The SL-BS was found to have good antifungal activity against *C. gloeosporioides, F. verticilliodes, F. oxysporum* f. sp*. pisi, Corynespora cassiicola,* and *Trichophyton rubrum*, offering eco-friendly antimicrobial agents for the agricultural sector.

Like bacteria and yeast, endophytic fungi are also one of the important sources to obtain novel bioactive compounds including BSs which can be utilized for varied biological applications. Some of the endophytic fungi have also been reported for the production of BSs. Adnan et al. (2018) isolated plant growth-promoting endophytic fungus *Xylaria regalis* from a coniferous tree *Thuja plicata*. Various parameters including the length of shoot and root, dry matter production, chlorophyll, nitrogen, and phosphorus contents in chili seedlings were improved in the presence of *X. regalis*. The BS-producing fungus also possesses antagonistic activity against *F. oxysporum* and *A. niger*, suggesting its potential applications in agriculture.

The rhizosphere of cereal crops such as wheat (*Triticum aestivum*) also provides a nutritionally rich environment to harbor diverse microbial communities. However, wheat, a staple food crop, is severely affected by powdery mildew disease. Powdery mildew, a foliar disease seen on grasses including cereals, is caused by the fungus *Blumeria graminis* f. sp*. tritici*. Symptoms of the disease show a powdery white to grey growth of fungi with spores on the surfaces of stems and leaves of wheat plants (Liu et al., 2015). It is difficult to control powdery mildew after its establishment. Therefore, strategies are employed to avoid the occurrence of infections at the early stages of the wheat crop. The early infection behaviors and occurrences of powdery mildew in wheat plants can be prevented through MELs. Yoshida et al. (2015) demonstrated the antifungal activity of MELs through an alteration in the hydrophobicity of the surfaces, where the authors used leaves of two Gramineae plants—wheat and rice—and two non-Gramineae plants—strawberry and mulberry—as model systems. MELs strongly reduced the hydrophobicity of solid surfaces of the leaves of wheat and rice as compared with strawberry and mulberry. The extent of the antifungal effect of MELs was dependent on the combination of fungal species and type of MEL-BS. MEL suppressed i) germination of conidia, ii) germ tube elongation, and iii) appressoria formation in *Blumeria graminis* f. sp*. tritici, Colletotrichum dematium, Glomerella cingulata,* and *Magnaporthe grisea*.

BS-based formulations are, therefore, an emerging and promising material to combat phytopathogens. BSs alone or as part of a formulation in combination with other biomaterials such as chitosan nanoparticles (CHNPs) of biological origin are considered to be beneficial for agricultural applications by improving soil quality and the degradation and/or solubilization of pesticides. BS-based biopesticides have remarkable features compared to chemical-based pesticides (Table 2). The use of BSs in biocontrol formulation could be a feasible approach to improve soil health and plant growth. These bio-based molecules possess immense potential to replace synthetic chemicals (Sangwan et al., 2022). The critical micelle concentration (CMC) is an imperative feature of surfactants, indicating the concentration of a BS or surfactant in the bulk phase. Above the CMC, surfactant molecules form aggregates which are termed micelles. Above the CMC, amphiphiles are typically seen as aggregates and monomers (Crouzet et al., 2020), where factors such as pH and temperature affect the CMC and activity of BSs (Zhong et al., 2015; Mnif and Ghribi, 2016). All these parameters subsequently affect the efficiency of BSs as “biopesticides.”

**TABLE 2:** Comparison between biosurfactant-based biopesticides and chemical-based pesticides.

Parameters	Biosurfactant-based biopesticides	Chemical-based pesticides	References
Synthesis and production from waste	Biological means: microbes and plants. Many low-cost and renewable substrates are utilized	Chemical means	Jarvis and Johnson (1949); Desai and Banat (1997); Rebello et al. (2020)
Production and downstream processing cost	High Produced as mixed congers	Comparatively low	Manga et al. (2021)
Toxicity and environmental friendliness	Environmentally safe	Comparatively higher toxicity	Edwards et al. (2003); Santos et al. (2016)
Hydrophobicity and aqueous solubility	Low and high	High and low	Satpute et al. (2010), Varjani and Upasani (2017)
Mode of action against pathogens Functional potential	Specific Versatile	Non-specific Restricted	Debode et al., (2007); Santos et al. (2016)
Applications in agriculture	Broad-spectrum	Narrow spectrum	Sachdev and Cameotra (2013); Karamchandani et al. (2022a), Chopra et al. (2020a)
Environmental stability	Comparatively high stability	Comparatively low stability	Jimoh and Lin (2019)
Concentration required for biological activities	Comparatively low	Comparatively high	Sachdev and Cameotra (2013)
Resistance developed by pathogens	Comparatively low due to the structural complexity of the molecules	Comparatively lower structural complexity of the molecules and frequent or repeated exposure to pathogens	Malakar and Deka (2021)
Challenges associated with the production process	• Complex production processes are involved in the production of biosurfactants from microorganisms• Processing of raw/or cheap substrates essential• Comparatively low yield	• Chemical synthesis process• Requires defined and predictable chemical substrates• Comparatively high yield	Mohanty et al. (2021)
Life cycle assessment (LCA)	• Comparatively more sustainable• Lower environmental impact• Less reports available—requires more attention	• Less sustainable• Comparatively higher environmental impact• More reports available	Manga et al. (2021); Kashif et al. (2022); Briem et al. (2022)

RLs derived from the genera *Pseudomonas* and *Burkholderia* exhibit antimicrobial activities against several pathogens. Substantial literature discusses the antimicrobial activity of RLs; however, few studies discuss the actual mechanisms through which RLs exhibit activity against pathogens and their overall effect on plant physiology. Platel et al. (2022) explored both, transcriptomic and metabolomic approaches, to reveal the mechanisms responsible for RL-BS-induced resistance of wheat crop against the hemibiotrophic fungal pathogen—*Zymoseptoria tritici*. Like maize and rice, wheat is also an economical and staple food worldwide. However, the quality and quantity of wheat grain are severely affected by the fungus *Z. tritici*, resulting in ∼50% in yield losses. Wheat plant was treated with bioinspired synthetic RL (mono, with a 12-carbon fatty acid chain: dodecanoyl α/β-L-rhamnopyranoside-Rh-Est-C12) under both test (infectious) and control (non-infectious) conditions to investigate its ability to provoke defense mechanisms. Rh-Est-C12 proved to be significant in protecting wheat plants from *Z. tritici*, as evident in the reduction of the disease severity by 41%. A noticeable observation was made by Platel et al. (2022) , who suggested a minor effect of RL on gene expression along with the accumulation of metabolites in wheat leaves. The authors also suggested the expression of approximately 24 differentially expressed genes (DEGs) and 11 differentially accumulated metabolites (DAMs) in wheat after treatment with Rh-Est-C12. Further bioassays also confirmed the antimicrobial activity of Rh-Est-C12 against *Z. tritici*. Research by Platel et al. (2020)and Platel et al. (2022) shed light on the means by which RLs affect the wheat phytopathogen *Z. tritici*.

## Improving the solubility and/or degradation of pesticides using biosurfactants

**FIGURE 3:**
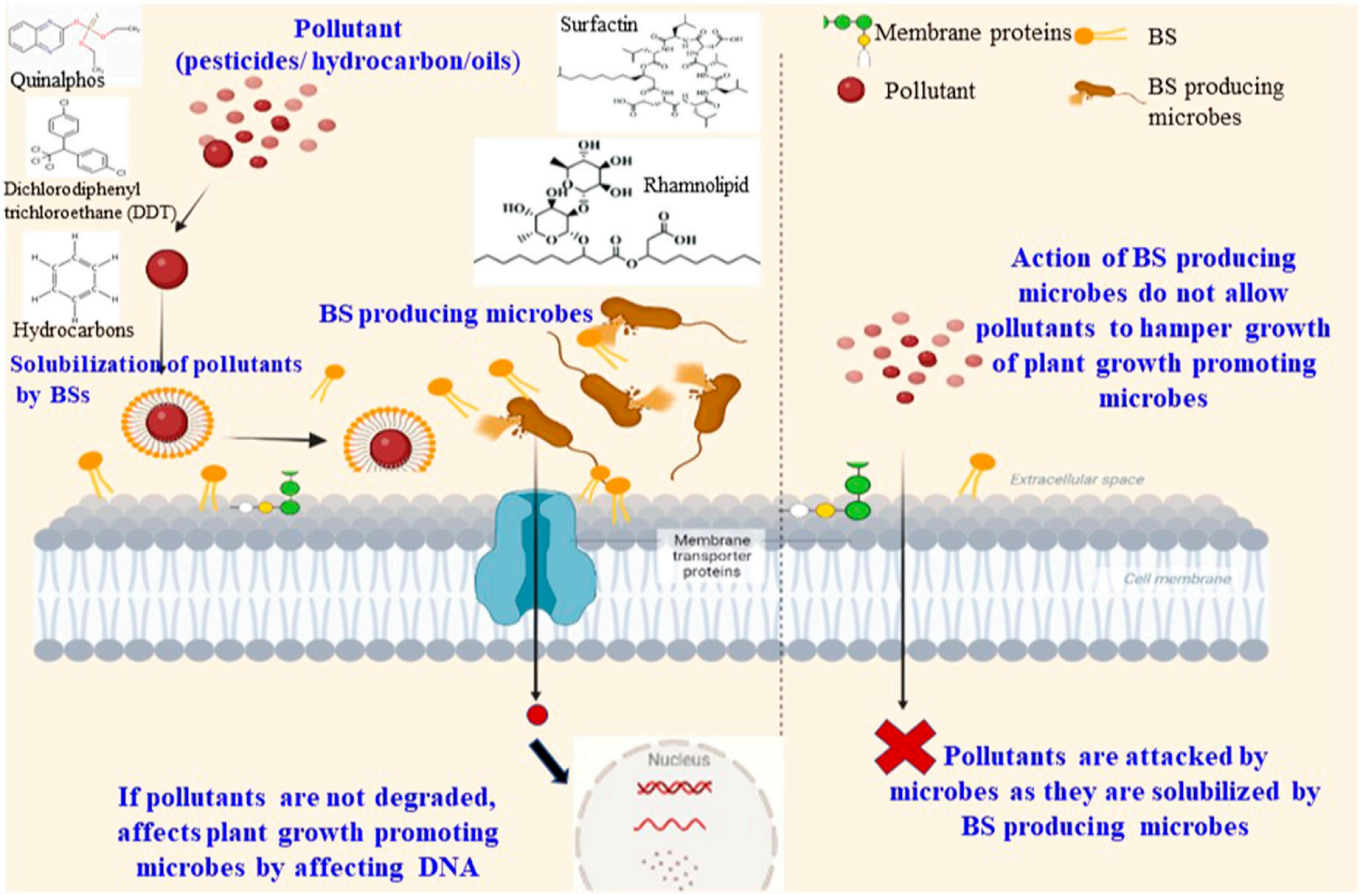
Mechanistic action of biosurfactants (BSs) on pollutant/s (pesticides, hydrocarbons, and crude oil).

The rampant use of pesticides during the green revolution led to a tremendous increase in food production. However, the repercussions are being endured by the current generations and will be dealt with by future generations, if proper measures are not undertaken to remove pesticides from the environment (Raj et al., 2021). Pesticides are extremely challenging to completely degrade and/or remove from the environment due to their poor aqueous solubilities and bioavailabilities. In general, the severe toxic effects of pesticides occur due to their high concentrations. Referring to the acute hazard ranking of insecticides, the World Health Organization (WHO) has identified quinalphos (an organophosphorus material) as moderately hazardous chemical. Despite the toxicity of quinalphos, they remain in use in agriculture. Approaches reported in the literature for pesticide removal involve the use of multifunctional molecules such as BSs, in addition to other chemicals either in pure or crude form. The use of microorganisms for the degradation and/or removal of pesticides or pollutants in order to reduce their concentrations is a wise approach (Figure 3). In pesticide formulations, the use of chemical surfactants (mostly of petroleum or petrochemical origin) has a negative impact on the environment and leads to hazardous effects on plants as well as the organisms associated with them. To avert these hazards, the alternative of using BSs possessing similar properties is a lucrative opportunity (Ali et al., 2022). BSs are very much capable of the dissolution or dislodging of hydrophobic pesticides and, thereby, increasing their bioavailability in the surroundings for remediation purposes. The issue of hydrophobicity and restricted pesticide accessibility can be tackled using BSs, where their amphiphilic nature facilitates effective interaction with pesticides.

Metabolically versatile BS-producing strains are usually efficient in solubilizing pesticides that are adhered to agricultural soils. Hassen et al. (2018) isolated the PGPR strain *Pseudomonas rhizophila* S211 from a pesticide-contaminated artichoke field, which grew in olive mill wastewater (OMWW)-based media. A molecular investigation of the strain revealed the presence and involvement of key genes in the synthesis of 1-aminocyphytopathogenscarboxylate deaminase, putative dioxygenases, auxin, pyroverdin, and exopolysaccharide levan. The strain S211 also produced RL-BS with a yield of 720.80 ± 55.90 mg/L, having an emulsification index (EI) of 90%, and an oil displacement of 63.58 cm^2^. The optimum conditions required for high BS production were 15% (v/v) OMWW, 40°C temperature, and 6.0 pH, with 0.5% (v/v) inoculum size and incubation for up to 8 days. The BS exhibited good stability over a wide temperature (40–90°C), wide pH (6–10), and NaCl concentration (up to 300 mM), suggesting its application potential in various conditions.

Gaur et al. (2019) used RL produced from *Lysinibacillus sphaericus* IITR51 for the dissolution of α- and β-endosulfan and γ-hexachlorocyclohexane. The BS significantly reduced the SFT of water from 72 to 52 N/m, with an EI of 48%. The BS also exhibited high stability over a wide pH (4.0–10), temperature (4–100°C), and salt concentration (2–14%). The RL efficiently dissolved α- and β-endosulfan as well as γ-hexachlorocyclohexane by up to 7.2, 2.9, and 1.8 fold, respectively, at a concentration of 90 mg/L BS. Additionally, the bacterium also had the potential to utilize aromatic organic compounds (benzoic acid, chlorobenzene, and 3- and 4-chlorobenzoic acid) and was resistant to heavy metals such as arsenic, lead, and cadmium.

The bioremediation of pesticide-polluted areas needs urgent attention due to their toxic effects. BSs possess high efficiency to solubilize contaminants or compounds compared with Tween 80, and a similar activity to SDS. Wattanaphon et al. (2008) reported the production of glucolipid BSs (molecular mass: 550.4 g/mol, yield: 6.5 ± 0.7 g/L, and CMC: 316 mg/L) from *Burkholderia cenocepacia* BSP3, an isolate from fuel oil-contaminated soil. The extracted BSs reduced the ST by up to 25 ± 0.2 mN/m, with good emulsion stability. The authors demonstrated the enhanced solubility of three pesticides (methyl parathion, ethyl parathion, and trifluralin) at BS concentrations above and below the CMC, and emphasized its potential role in surfactant-enhanced remediation (SER) to clean polluted sites. Biodegradation of some organochlorine and organophosphate pesticides is challenging due to their lower solubilities and bioavailabilities. Generally, surfactants at concentrations below their CMC do not effectively solubilize solutes, usually related to the properties and concentration of the surfactant. The solubility of organic compounds such as dichloro-diphenyl-trichloroethane (DDT) can be increased by using synthetic surfactants even at concentrations below their CMCs. These activities are achieved due to the partition-like interaction of solutes, facilitating mixing between immiscible solutions at equilibrium (Kile and Chiou, 1989). Thus, BSs promote the solubility of water-insoluble organic solutes below their CMC, while above the CMC, hydrophobic compounds are strongly solubilized due to the aggregation or accumulation of the micelle structure of the surfactant molecule (García-Reyes et al., 2018). Glycolipid BSs display better solubilization of pesticides than synthetic surfactants. To overcome the hydrophobicity issue of pesticides, García-Reyes et al. (2018) reported the effective role of a glycolipid BS in enhancing the solubility of endosulfan (from 0.41 to 0.92 mg/L) and methyl parathion (from 34.58 to 48.10 mg/L). The BS produced from *Pseudomonas* sp. B0406 reduced the ST by up to 40.4 mN/m at the CMC (1.4 g/L).

The poisonousness of the organophosphorus insecticide quinalphos is quite high and, like most other insecticides, it is sparsely soluble in water due to the presence of a chloride radical, which appears to hinder their degradation. For the bioremediation of such compounds, Nair et al. (2015) successfully isolated an autochthonous bacterium that utilizes quinalphos as a carbon source for its growth. The authors carried out initial screening assays to enrich and isolate pesticide-degrading bacteria (12 isolates) from contaminated soil. The authors demonstrated the degradation of pesticide-contaminated soil by approximately 86, 82, and 94% by *Pseudomonas* sp., *Serratia* sp., and *P. aeruginosa*, respectively. These bacteria were capable of utilizing high concentrations of quinalphos as a carbon source in a shorter incubation period*. P. aeruginosa* rapidly degraded quinalphos to 2-hydroxy quinoxaline and phosphorothioic acid even at high concentrations due its ability to produce BSs. The lower bioavailabilities and higher toxicities of endosulfan and endosulfate hampered the bioremediation of contaminated soil. Odukkathil and Vasudevan. (2015) reported BS-producing strains of *Bordetella petrii* (I GV 34 and II GV 36) for the biodegradation of endosulfan and its isomers. The BS produced by the strain reduced the ST by up to 44 mN/m. The authors also reported the degradation of isomers α (89%) and β (84%) by *B. petrii* I GV 34, whereas *B. petrii* II GV 36 degraded 82% of both isomers. Such studies are imperative in solubilizing pesticides at higher concentrations.

Some agricultural, cheap substrates have been utilized for agrochemical formulations to extract BSs and even to solubilize chemical pesticides. The research conducted by López-Prieto et al. (2020) demonstrated the use of corn steep liquor (CSL) for the extraction of a BS (rich in lipopeptides) and its utilization in an agrochemical formulation. CSL is a by-product of corn wet-milling, where corn kernels are broken into various components such as corn oil, protein, corn starch, and fiber. This study demonstrated a reduction in the concentration of copper oxychloride (Cu-Oxy)—a broad-spectrum fungicide. The low aqueous solubility of Cu-Oxy hinders its penetration in the fungal mycelial network, leading to a loss of efficiency as a fungicide. The BS extracted from CSL was incorporated as an additive which led to the enhanced aqueous solubility and efficiency of the Cu-Oxy fungicide. A comparative study using Tween 80, SDS, and cetyltrimethyl ammonium bromide (CTAB) reported the dissolution of Cu-Oxy by up to 90% (4 g/L) within 20 min of contact with the BS (20 g/L). The amphoteric nature of the lipopeptide BS facilitated Cu-Oxy solubilization and the effective formation of a complex (BS–pesticide micelles) with water molecules, when compared with synthetic surfactants. The highest solubilization (96.5%) was achieved within 20 min when the BS (16.1 g/L) and Cu-Oxy (2 g/L) were used, while <0.5% dissolution of Cu-Oxy (4 g/L) was observed when synthetic surfactants were used. Findings from such studies are significant in solubilizing chemical pesticides through the usage of BSs.

Further investigations are certainly needed to promote green technology through the utilization of environmentally safe substrates in the production of BSs. For example, Gómez-Graña et al. (2017) extracted a novel BS from CSL and utilized it to further the green synthesis of metal, gold, and silver NPs using a one-step procedure which was initiated by temperature treatment. It is important to highlight that most of the BSs were produced from pathogenic microbes. The authors extracted lipopeptide BSs from a fermented liquid stream of CSL using lactic acid bacteria (LAB) and used them to further the green synthesis of metal NPs. The BSs facilitated the stabilization of the NPs, which enhances their inhibitory activity against pathogens. The BSs were helpful in reducing metal precursors and efficiently stabilizing the NPs for antimicrobial activity against *E. coli*, *P. aeruginosa*, and *S. aureus*. Such studies demonstrate the potential use of BSs with NPs for broader applications. The BS-stabilized NPs effectively inhibited *E. coli*, compared with citrate-stabilized silver NPs. Such studies are significant for reducing environmental impacts, minimizing waste, and enhancing the energy efficiency of nanomaterials for a wide range of applications. Another study reported by Joanna et al. (2018) suggested the use of *B. subtilis-*derived BS in the stabilization of NPs. The solubilized NPs possess a better antimicrobial activity, or act as an enhancer for biogenic AgNPs through a non-specific synergistic effect. Kiran et al. (2010) reported the stability of silver NPs, with uniform morphology, when they were synthesized using glycolipids of *Brevibacterium casei* MSA19, grown in agro-industrial and industrial waste, as the substrate. Farias et al. (2014) demonstrated the use of BSs of *P. aeruginosa* origin in the synthesis of spherical-shaped silver NPs using a microemulsion technique.

Exploring nanotechnology in the delivery of pesticides is a comparatively innovative approach and is still under development. The main focus of this approach is reducing the indiscriminate usage of pesticides and ensuring their application in the field in a safer way. Nanoencapsulation process and nano-encapsulated pesticide formulation for the delivery of pesticide.

The advancement of biodegradable nano-encapsulated pesticide formulations has improved properties such as permeability, solubility, stability, and even specificity. The protection from degradation of active components of pesticides, along with enhancing their long-term efficacy against pests can be achieved using a nanoencapsulation approach. Additionally, nanoencapsulation allows for a reduction of the actual dose of pesticides as well as reduced direct exposure to human and animals, while protecting crops from pests (Nuruzzaman et al., 2016). Intense research efforts are necessary to reveal the mechanisms responsible for the synthesis of nano-encapsulated pesticide formulations. Further detailed investigations of these materials’ behavior in plant systems and the environment would facilitate the establishment of guidelines and a regulatory framework for their commercialization. Agro-research has been focused toward designing and developing organic NP-based formulations. Nanotechnology has substantially contributed toward developments for sustainable agriculture (Prasad et al., 2017; Singh et al., 2021). International commercial organizations, such as Syngenta, are marketing various microemulsion products (Karate ZEON, Subdue MAXX^®^ Banner MAXX^®^) for agricultural applications. Nano-pesticides typically incorporate metal and metal oxide NPs such as titanium dioxide, silica, zinc, iron, and gold nano-rods. The release of these metals is non-toxic to the soil and helps in eliminating pests and improving the soil quality. The fungicide MAXX^®^ provides protection (from contact and systemic infections) to the individual plant. Warm or humid weather-associated infections such as pythium blight, yellow tuft (downy mildew), and pythium damping-off can be controlled by using fungicide MAXX^®^. Alternatively, a more biocompatible strategy encompasses the formation of NPs using fungi (Patil et al., 2021; Karamchandani et al., 2022a).

Zygomycete fungi are a rich source of biopolymer CH. This biomaterial possesses exceptional antimicrobial potential and also acts as a source for synthesizing CHNPs. Chandra et al. (2015) synthesized CHNPs from CH (commercial source) and amended them to a nano-delivery system to induce innate immunity in plants. Recently, our research group (Karamchandani et al., 2022a) explored the antimicrobial potential of RL-BS (commercial) in combination with fungal chitosan (FCH from *Cunninghamella echinulata* NCIM 691) and FCH-derived NPs (FCHNPs). Both antibacterial and antifungal assays conducted against phytopathogens proved the antimicrobial potential of the three test compounds. The MICs of RL-BS (256 μg/ml), FCH, and FCHNPs (>1,024 μg/ml) were higher against *Xanthomonas campestris* NCIM 5028 when they were used singly. A reduction in the MIC by up to 128 and 4 μg/ml for RL-BS with FCHNPs was observed in combination studies, whereas the MIC for RL-BS with FCH was also reduced by up to 128 and 256 μg/ml, respectively. The test compounds displayed dose-dependent antifungal activity and inhibited fungal spore germination (61–90%). Thus, combination studies using RL-BS with FCHNPs support the suitable development of eco-friendly and low-cytotoxic formulations for agricultural outlooks. BSs are certainly useful in developing nano-formulations for sustainable and eco-friendly agricultural practices (Ozdal et al., 2022). Figure 2 shows BS-based eco-friendly applications in the management of diseases (bacterial and fungal) and the remediation of engine oil (heavy)-contaminated soil. The physicochemical and functional properties of BSs are crucial for designing a BS-based formulation for agricultural purposes.

## Role of biosurfactants in improving soil quality through the removal of crude oil, hydrocarbons, and metals

Followed by applications of BSs against phytopathogens, the solubilization and/or degradation of pesticides and the removal of water-insoluble complex contaminants (crude oil, petroleum hydrocarbons, crude oil, and heavy metals) are crucial to improve the quality of agricultural soil. Environmental and ecological pollution caused by pollutants can be treated using the high activities of BSs or BS-producing organisms. Most contaminating pollutants bind firmly to soil particles through strong sorption and hydrophobic interactions, making it difficult to separate or remove them from the environment (Figure 3). Microbial surfactants or BSs can dislodge these contaminants or pollutants from soil particles by emulsification, solubilization, or mobilizing activities, as well as by reducing the ST and IFT. The emulsification of BSs creates an increased surface area of crude oils/hydrocarbons, enhancing the solubility in aqueous environments. The BS molecules bind to the hydrocarbons/crude oils *via* their hydrophobic chains, ultimately forming hydrophilic bonds with nearby water molecules. Such effects can be accomplished through augmentation of the BSs or BS-producing active microbes to the contaminated soil samples or environments. The literature discusses the stimulating or positive effect of BSs on the biodegradation of hydrocarbon pollutants in the environment. Thus, BSs facilitate the solubilization and/or removal of hydrophobic substances, complex hydrocarbons, and crude oils from soil and sand particles (Jahan et al., 2020; Yesankar et al., 2022). BSs have been widely reported for solubilization and/or remediation of pollutants to improve the quality of agricultural soil (Sachdev and Cameotra, 2013). The diversity, flexibility, and eco-friendly nature of BSs have generated particular interest in agriculture for bioremediation-related projects (Bami et al., 2022).

Cyclic lipopeptide surfactins exhibit powerful emulsifying activities and, therefore, have been utilized in the remediation of oil-contaminated soil. Phulpoto et al. (2020) isolated a BS-producing *B. nealsonii* S2MT from lake sediment in China and presented a number of notable physical properties of the BS. Surfactin reduced the ST by up to 34.15 ± 0.6 mN/m with the emulsification of kerosene (55 ± 0.3%). The authors further demonstrated the abilities of surfactin in the remediation of heavy engine oil-contaminated soil by up to 43.6% and 46.7% at concentrations of 10 and 40 mg/L, respectively, proving its suitability for applications in the environment (Phulpoto et al., 2020). A combination of hydrocarbon-degrading and BS-producing bacteria is suitable to remove crude oil contamination. Chen et al. (2020) used the BS-producing, salt-tolerating, and crude oil-degrading bacteria *Dietzia* sp. CN-3, and *Acinetobacter* sp. HC8–3S for the removal of contaminants. The efficient bacterial consortium could degrade crude oil, achieving a 95.8% degradation efficiency in 10 days at a pH ranging between 4 and 10 and salinity conditions between 0–120 g/L. Crude oil, containing various n-alkanes, cycloalkanes, branched alkanes, and aromatic hydrocarbons, was also well-degraded when compared to the individual strain. Moreover, cloning experiments carried out for two alkane hydroxylase genes (alkB in CN-3 and alkM in HC8–3S), and real-time quantitative polymerase chain reactions (PCR), showed a prominent expression of the alkB gene in the utilization of hydrocarbons containing long-chain alkanes (C20, C24, and C26), as well as alkM in the degradation of medium and long-chain alkanes (C14, C16, C20, C24, and C26).

An eco-friendly disposal and/or recycling process for metal or metalloid contaminants is essential for the mining industry. The application of crude BS was confirmed by Lopes et al. (2021) , who developed a rapid method for the precipitation of metal. The BS-mediated remediation of soil samples that are contaminated with metals is an innovative approach in the mining industry, where the disposal and recycling of metal contaminants can be achieved. The authors used a mixture of RL (di-congeners: 85%) extract from *P. aeruginosa*, grown in a glycerol-containing medium, and evaluated its effect on sandy soil which was artificially contaminated with hydroxydecanoyl-hydroxydecanoate of short- and long-term sites. RL, being anionic in nature, binds strongly to metals (cadmium and zinc) with a high extractive capacity (for transition metals and metalloids), and removed arsenic (53%), cadmium (90%), and zinc (80%) from artificially contaminated soil. Another aspect of the study also demonstrated the exceptional biocompatibility of BSs with *Artemia salina—*a brine shrimp (aquatic crustacean). Additionally, a growth inhibition assay was carried out to demonstrate the effect of RL-BS on commensal bacteria and yeast. This comprehensive study suggested a broader perspective on recycling metal contaminants, along with reducing monetary inputs required for mining activities. This research also reinforced the conceivable, eco-friendly approach for soil remediation processes.

Other phytoremediation approaches involving the use of plant extracts were reported for the degradation of contaminants. Wang et al. (2017) studied the phytoremediation of agricultural soil contaminated with dichloro-diphenyl-trichloroethane (DDT) using various preparations containing BS-producing *Pseudomonas* sp. This work showed an enhanced bioavailability and an improved degradation of DDT. The removal efficiency of all the different preparations was satisfactory and ranked as follows: fertilizer + perennial ryegrass (69.0%) > fertilizer + perennial ryegrass + *Pseudomonas* (65.9%) > fertilizer + tall fescue + *Pseudomonas* (65.6%) > fertilizer + tall fescue (59.4%) > the fertilizer control (40.3%).

## Life cycle assessment and life cycle sustainability analysis of biosurfactants

The wide-ranging applications of BSs in the agricultural sector come with a variety of issues/challenges and environmental risks. The use of BSs in bioremediation and biodegradation aids in the removal of the pollutants; however, the environmental impact of BSs needs to be evaluated in order to determine their overall impact (Manga et al., 2021). Numerous aspects such as production, distribution, and end-use of BSs should be well-planned before convincingly establishing their sustainability. Currently, literature dealing with these issues is limited, and the use of BSs as sustainable products in the framework of societal, commercial, and environmental aspects requires focused attention.

Several researchers have investigated the production and utilization of BSs in an environmentally sustainable way. Life cycle assessment (LCA) and life cycle sustainability analysis (LCSA) have recently been reported as an means to quantify the impact of human activities/interventions from environmental, social, and economic perspectives. These three concepts or models are well-linked and accepted by the United Nations (UN) as a base to propose sustainable development goals (SDGs) (Purvis et al., 2019). SDGs solely endorse the transition from the usage of non-renewable resources to renewable resources, in order to improve all contributions, including the value of products. Even though chemically-derived or synthetic surfactants have attained an indispensable importance, they originate from petrochemical and oleochemical compounds, which are non-renewable sources. Current research focuses on the use of BSs as sustainable alternatives to synthetic surfactants.

The LCA protocols evaluate the processing, from the initial stages (raw materials, production, distribution, etc.) to the final stages (application, recycling, and ultimate environmental fate), of the product, including end-of-life and disposal (Kashif et al., 2022). Few other modes of assessment similar to LCA take into account certain forms of inputs such as capital cost, infrastructure, energy, or gains throughout the process. Using this information, the net impact of the process is quantified. The LCA framework includes a defined goal and scope, as well as analysis of the inventories’ impact and interpretation (Manga et al., 2021). BS has been used at different stages in agricultural activities, and LCA and LCSA would be beneficial in assessing their impact at each stage, in order to establish their sustainabilities. A study conducted by Rebello et al. (2020) revealed that the LCA of BSs presented a lower environmental impact than other synthetic detergents. Thus, synthetic surfactants should be avoided, and further investigation on the production of BS-based formulations should be encouraged as a first step toward environmental sustainability. Aru and Ikechukwu (2018) assessed the impact of the production of BSs from oil waste using two cultures, namely, *Azotobacter vinelandii* and *Pseudomonas* sp., using the International Organization for Standardization (ISO) 14040 standard. Such standards facilitate the quantitative assessment of different environmental aspects of a proposed product or intended service, at different stages of its life cycle. Aru and Ikechukwu (2018) concluded that a process involving a di-culture approach is more sustainable than individual cultures. Following ISO 14040, Kopsahelis et al. (2018) carried out a comparative LCA of SLs and RLs. During the synthesis of these BSs, the authors found that air emissions, thermal requirements, and electricity are crucial decisive factors for sustainable practices. The authors also observed that the environmental impact of the synthesis of SLs was 22.7% higher than that of RL synthesis (Kopsahelis et al., 2018). The LCSA approach utilizes an amalgamation of diverse model frameworks to resolve specific challenges. However, limitations, such as choosing and configuring existing models, are major concerns that must be addressed when undertaking a challenge or solving a problem. LCA has therefore been considered as the most appropriate method, in terms of guidelines, policies, and regulations related to the ISO body, to provide appropriate methods and protocols**.**

## Conclusion

The disproportionate use of pesticides and agrochemicals for improving crop yields adversely affects all biota on Earth. Many studies have linked chemical pesticides with biological disorders as a result of modulation of genes. BSs offer broader agricultural perspectives by combating phytopathogens and improving soil quality through enhanced solubilization/degradation of pollutants such as crude oil, hydrocarbons, heavy metals, metalloids, and pesticides. The antimicrobial/biocontrol activities of BSs allow their use as “biopesticides” to manage diseases in economically valuable crops. The extraordinary physicochemical and functional properties of BSs and BS-producing microorganisms possessing plant growth-promoting traits are favorable for the agriculture sector due to their biodegradabilities, biocompatibilities, antimicrobial actions, and low cytotoxicity effects. RLs and surfactin/lipopeptides have been frequently compared with MELs, SLs, TLs, and CL for crop protection, consequently encouraging the scientific fraternity to explore other amphiphilic molecules for agricultural perspectives. LCA and LCSA allow analysis of the environmental impact of BSs derived from various renewable substrates by microbial cultures, including product systems, products, services, and processes, which are essential. BS-based eco-friendly formulations in combination with other biomaterials or nanoparticles may represent a vital component for a safe agro-industry and greener future.

## Future prospects, market concerns, acceptance, and challenges

The overuse and misuse of agrochemicals against phytopathogens have negatively affected the agricultural sector. Agricultural productivity needs continuous expansion to meet the demands of the ever-increasing population. BS, in the form of a biocontrol formulation, represents a potentially conceivable approach to improve plant health. The antagonistic properties of BSs against several phytopathogens can be explored and subsequently used to develop biocontrol formulations to protect crops. Recent reports on the combination of BSs with nanotechnology seem to be a more attainable approach for developing innovative strategies in crop improvement. Developing efficient disease strategies is possible through understanding the genetic make-up of the pathogen, combined with an in-depth knowledge of resistance genes and the virulence structure of pathogens. Advanced tools such as metagenomic and *in silico* are quite supportive in characterizing pesticide or pollutant-degrading microorganisms, up to the taxonomic and functional level. The role of BSs and the mechanisms involved in the bioremediation of pesticides warrants detailed investigation. BSs, as bio-stimulant and biocontrol agents that enhance plant–bacterial interactions, require further exploration.

BSs have an obvious edge over their synthetic counterparts; however, major concerns are the production costs and the overall yields, which are quite low for most BSs. The low profit-to-investment ratio makes sustainable BS production challenging, as well as the marketing of BSs at cost-effective prices (compared to synthetic surfactants). Various studies have presented different strategies and approaches to resolve such challenges, and to enhance commercial feasibility. BSs are significant components of the worldwide market. Rigorous regulations are mandatory for using BSs in various applications. Analysis by the business assessing/consulting firm Grand View Research ^3^ (2015) suggested that in the year 2013, the BS market was approximately 344,068.40 tons, and was anticipated to reach approximately 461,991.67 tons (in 2020) with a growth of 4.3% (from 2014–2020). The revenue generated by the BS market is enormous (> USD 1.8 billion in 2016) and is further predicted to increase up to USD 2.6 billion by the year 2023. As per the Global Markets Insights (2018)^4^ observations, the RL market has expanded by 8%. One of the latest studies by the Global BS market^5^ predicted an increase up to USD 1,442.7 million (by 2026) from USD 1,375.4 million (in 2020), at a compound annual growth rate (CAGR) of 0.8% from 2021–2026 (Global Biosurfactants Market Outlook 2021). These figures certainly confirm the impact and promise of BSs in the global market.

Regardless of the cumulative worldwide demand, limitations in cost competitiveness of these surface-active molecules are still the foremost concern. The inclusive cost breakdown for the production of a BS, or any other biological product, encompasses the valuation of capital and operating expenses, which are dependent on the size as well as number of process equipment, raw materials required, consumables, supporting utilities, labor, facility-dependent items, waste treatment and disposal, and additional resources utilized (Petrides 2003; Harrison et al., 2015). Significant variation in the capital cost investment seems to be dependent on the type of intended BSs, equipment, and the annual target. For example, the production of surfactin requires elaborate downstream processing and purification using high-performance liquid chromatography (HPLC) along with solvent extraction, whereas for SL type BS production, only the solvent extraction process need be carried out. Stainless steel-based vessels cost approximately 2.5–3 times more than a carbon steel vessel of similar capacity. A titanium-based vessel costs approximately 15 times more than a carbon steel vessel (Petrides 2003; Harrison et al., 2015). With the ever-increasing demand for BSs, scientific fraternities are nowadays exploring low-cost and renewable substrates, or waste materials, aiming to reduce the manufacturing costs. The utilization of various low-cost materials will significantly decrease the cost required for BS production. Various feedstocks have been utilized in the production of BSs, providing financial benefits and adopting environmental applications requiring minimal or reduced downstream processing treatments, as opposed to other more demanding health or biomedical applications.

The success story of “Serenade ASO ^6^ (bio-fungicide)—a bacterium-based commercial product” is encouraging for the future outlook of BSs and their market significance. “Serenade ASO” is an authorized and marketed “bio-fungicide” produced by Bayer CropScience Ltd. (Cambridge, UK). It is a foliar fungicide that contains a suspension of *B. subtilis* QST 713, along with its fermentation residues and water. The foliar formulation is useful for reducing damage caused by fungal diseases in a variety of agricultural and horticultural crops. The mechanistic action includes the creation of a zone of inhibition on the plant leaf, which prevents attachment and penetration of the pathogen. The biological compounds produced by the bacterium destroy germ tubes and mycelial networks of fungal pathogens by puncturing their cell membranes.

Some of the big players, viz., 1. BASF-Cognis based in Germany and the USA, and 2. Ecover in Belgium have contributed to the manufacture of BSs. Other contributors such as Jeneil Biosurfactants (Saukville, Wisconsin, USA) have created a prominent footprint in the BS area. RLs are being commercially produced by AGAE Technologies LLC, USA and Rhamnolipid Holdings Inc. (New York, USA). Fraunhofer IGB, a German-based company produces MEL. Other industries, namely, Saraya from Japan, Ecochem Ltd from Canada, Intobio from South Korea, and Sigma-Aldrich Co. from the USA, are prominent in the BS market (Dhanarajan and Sen 2014; Ashby et al., 2013). SL produced by SophoronTM and sold by Saraya (Japan), and surfactin by Soliance (France) and Holiferm (UK) are also popular. RAG-1 emulsan is produced by Petroferm Research Inc. Rl and vended by Ecover (Boulogne-sur-Mer, France), which are noticeable contributors in the global BS market. Finally, Unilever partnered with Evonik and launched a green, dishwashing product “Quix” in 2019; the first RL in the world used in a household cleaning product, establishing their potential use in detergent formulations.

Not everything goes smoothly in the BS manufacturing and commercialization process. Challenges, such as the creation of heavy foaming during processing of particular batches, availability of inexpensive, cheap, and renewable raw materials, low yields, and expenditure requirements for downstream processing and purification protocols, are faced at the industrial BS production level (Bertrand et al., 2018; Schultz and Rosado, 2020). Efforts to reduce BS production costs include the use of waste from various industrial sectors such as food, agricultural, and oil processing as substrates, offering low-cost materials (Banat et al., 2014; Pele et al., 2019; Marques et al., 2020). Additionally, many statistical tools are also used to reduce the costs of BS production. Streamlining these approaches will certainly facilitate economically feasible, universal BS production.

The combination of NPs of biological origin with microbial surfactants is recommended for use against phytopathogens to achieve greener agricultural practices. This new branch of “myco-nanotechnology” offers significant opportunities for several non-toxic applications and greener alternatives, compared with chemically synthesized NPs for agriculture. Nanotechnology is an extremely fast-growing branch of science, including the synthesis and development of numerous multifunctional nanomaterials for agriculture purposes. The synthesis of NPs from bacteria and fungi, or the inclusion of BSs in their synthesis requires much more attention in order to utilize this combination for various industrial applications, including agriculture. The synthesized NPs can be utilized for the detection and biocontrol of phytopathogens, particularly from an agricultural perspective. In this new era of agro-practices, the prime focus of research should be on designing and developing novel bioformulations comprising nanocarrier systems to improve the overall soil texture and health.

## Footnotes

1
https://www.who.int/publications/i/item/9789240005662.

2
https://www.businesstoday.in/latest/world/story/eu-commission-proposes-cutting-pesticides-by-50-by-2030-338740-2022-06-22.

3
https://www.grandviewresearch.com.

4
https://www.gminsights.com/industry-analysis/biosurfactants-market-report prnewswire.com/.

5
https://www.prnewswire.com/.

6
https://www.cropscience.bayer.us/products/fungicides/serenade-aso.

## Abbreviations

ACCD, 1-aminocyclopropane-1-carboxylate deaminase; APF, acid-precipitated fraction; BSs, biosurfactants; CHNPs, chitosan nanoparticles; CL, cellobiose lipids; CMC, critical micelle concentration; CSL, corn steep liquor; CTAB, cetyltrimethyl ammonium bromide; DDT, dichloro-diphenyl-trichloroethane; FCH, fungal chitosan; IAA, indole acetic acid; IFT, interfacial tension; LAB, lactic acid bacteria; LCA, life cycle assessment; LCSA, life cycle sustainability analysis; MELs, mannosylerythritol lipids; MIC, minimum inhibitory concentration; NPs, nanoparticles; PCR, polymerase chain reaction; PGPR, plant growth-promoting rhizobacteria; RL, rhamnolipid; SDGs, sustainable development goals; SDS, sodium dodecyl sulfate; SER, surfactant-enhanced remediation; SL, sophorolipids; ST, surface tension; TL, trehalose lipids.
